# Comparative efficacy and toxicity of Axicabtagene Ciloleucel *versus* Tisagenlecleucel in European patients with large B-cell lymphoma: a systematic review and meta-analysis

**DOI:** 10.7717/peerj.21410

**Published:** 2026-07-06

**Authors:** Haoxuan Li, Yingchu Liu, Tianyao Wang, Kunjiang Zhong, Jingyi Xiao, Xinyang Huang

**Affiliations:** 1 School of Basic Medicine, Capital Medical University, Beijing, China; 2 Beijing Anzhen Hospital, Capital Medical University, Beijing, China; 3 Beijing Shijitan Hospital, Capital Medical University, Beijing, China; 4 Peking University Third Hospital, Beijing, China

**Keywords:** Axicabtagene ciloleucel, Tisagenlecleucel, Large B-cell lymphoma, Europe, Efficacy, Toxicity, Chimeric antigen receptor T-cell therapy

## Abstract

**Background and Objectives:**

Large B-cell lymphoma (LBCL) is a common and aggressive non-Hodgkin lymphoma (NHL) characterized by abnormal proliferation of mature B lymphocytes, with a 10-year prevalence of up to 45 cases per 100,000 individuals in European populations and a continuing upward trend. Axicabtagene ciloleucel (Axi-cel) and Tisagenlecleucel (Tisa-cel) are the two most established chimeric antigen receptor T-cell (CAR-T) therapy products for LBCL, yet a systematic comparison of their efficacy and safety in European populations has been lacking. This systematic review and meta-analysis aims to address this gap by comprehensively evaluating differences in treatment efficacy and adverse events between Axi-cel and Tisa-cel in European patients with LBCL.

**Materials and Methods:**

We searched PubMed, Cochrane Library, Scopus, and other databases for studies published between January 2020 and February 2026, ultimately including eight European cohort studies with a total of 2,178 patients. Efficacy and survival outcomes comprised 3-month overall response (OR), 3-month complete response (CR), 12-month progression-free survival (PFS), and 12-month overall survival (OS). Toxicity outcomes included 12-month non-relapse mortality (NRM), all-grade and grade ≥ 3 cytokine release syndrome (CRS), all-grade and grade ≥ 3 immune effector cell-associated neurotoxicity syndrome (ICANS), all-grade and grade ≥ 3 neutropenia, thrombocytopenia, and anemia, as well as tocilizumab use and ICU support. A random-effects model was used for pooled analysis, with sensitivity analyses performed for outcomes exhibiting substantial heterogeneity.

**Results:**

Regarding efficacy, Axi-cel was associated with significantly higher 3-month OR and 3-month CR rates compared with Tisa-cel, whereas 12-month PFS was lower in the Axi-cel group. In terms of toxicity, the incidences of all-grade CRS, all-grade ICANS, and grade ≥ 3 ICANS were significantly higher with Axi-cel, and tocilizumab use was more frequent. No statistically significant differences were observed for the remaining outcomes.

**Conclusions:**

In European patients with LBCL, Axi-cel demonstrated superior short-term efficacy compared with Tisa-cel, but showed inferior performance on certain intermediate-term efficacy endpoints and was generally associated with greater toxicity and higher healthcare resource utilization. Clinical selection of a CAR-T regimen should be individualized, with careful consideration of efficacy, toxicity, and cost. Future high-quality studies with longer follow-up are needed to evaluate long-term outcomes and to validate the findings of this analysis.

## Introduction

Large B-cell lymphoma (LBCL) is a common, highly aggressive non-Hodgkin lymphoma (NHL) (Streich et al., 2023) arising from abnormally proliferating mature B lymphocytes in lymph nodes or extranodal tissues (Chaudhry et al., 2023). It encompasses several histological subtypes, including diffuse large B-cell lymphoma (DLBCL), primary mediastinal B-cell lymphoma (PMBCL), and follicular lymphoma (FL) (Bethge et al., 2022 among others) DLBCL is the most prevalent, accounting for approximately 40% of all NHL cases. (Coelho et al., 2024) DLBCL imposes a substantial global disease burden, with an annual incidence of 2.3–13.8 cases per 100,000 individuals (Garg et al., 2022); in Europe, the annual incidence in Germany alone is approximately seven cases per 100,000 (Skalt et al., 2022), and the overall 10-year prevalence across European countries is projected to reach 45 cases per 100,000, with a continued upward trend in the coming years (Kanas et al., 2022). Pathologically, DLBCL is characterized by the diffuse growth of malignant B cells, dysregulation of multiple molecular pathways including NF-*κ*B and PI3K/Akt/mTOR, and high mitotic activity with marked invasiveness and migratory potential.(Almasmoum, 2024) Patients with DLBCL typically present with rapidly enlarging lymphadenopathy, extranodal masses, and B symptoms; if left untreated, the median survival is less than one year, posing a serious threat to patient survival (Adıyaman et al., 2019; Flowers, Sinha & Vose, 2010; Pacis et al., 2024).

Chimeric antigen receptor T-cell (CAR-T) therapy is an adoptive cellular immunotherapy in which a patient’s own T cells are genetically engineered to express a CAR and then reinfused, enabling them to specifically recognize target antigens and eliminate tumor cells (Rababah et al., 2023). Although the immunophenotype of LBCL is highly heterogeneous, the majority of malignant B cells express CD19, a canonical B-cell marker. CD19-targeted CAR-T therapy has therefore been increasingly developed and applied in clinical practice (El Hussein et al., 2023). To date, the U.S. Food and Drug Administration (FDA) and the European Medicines Agency (EMA) have approved three CD19-targeted CAR-T products for the treatment of relapsed/refractory large B-cell lymphoma (R/R LBCL): Axicabtagene ciloleucel (Axi-cel), Tisagenlecleucel (Tisa-cel), and Lisocabtagene maraleucel (Liso-cel) (Hoffmann et al., 2023). Among these, Axi-cel and Tisa-cel have the most extensive clinical experience and the richest body of evidence for LBCL. The CAR constructs expressed by engineered T cells differ slightly between the two products: Axi-cel uses a CD28 costimulatory domain, whereas Tisa-cel employs a 4-1BB costimulatory domain (Smith & Shen, 2023); however, both incorporate an anti-CD19 single-chain antibody fragment, thereby ensuring their anti-LBCL activity. In both clinical trials and real-world settings, Axi-cel and Tisa-cel have demonstrated substantial efficacy. The ZUMA-1 study demonstrated, with five years of follow-up, that overall survival (OS) with Axi-cel in patients with refractory LBCL was 42.6% (Neelapu et al., 2023). The ELARA study reported a complete remission (CR) rate of 69.1% and an overall response (OR) rate of 86.2% with Tisa-cel in patients with relapsed/refractory FL (Fowler et al., 2022).

Although prior studies have compared the efficacy and adverse events of Axi-cel and Tisa-cel in LBCL, the majority of the literature derives from small, single-center cohort studies, making it difficult to draw broadly applicable conclusions. Existing meta-analyses comparing the two products remain limited in the comprehensiveness and rigor of their clinical assessments, and population-specific analyses in European patients—particularly regarding toxicity and adverse events—are still lacking. To address this, we conducted an updated meta-analysis integrating data from multiple European cohort studies, representing the first systematic evaluation of the comparative efficacy and safety of Axi-cel and Tisa-cel for LBCL specifically in a European population. Compared with prior meta-analyses, our analysis incorporated a broader set of safety endpoints to provide a more comprehensive assessment. The findings of this study are intended primarily to inform clinical decision-making for LBCL management in Europe, and may also serve as a reference for the implementation of CAR-T therapy in other regions.

## Materials and Methods

### Criteria for searching literature

This study followed the Preferred Reporting Items for Systematic Reviews and Meta-Analyses (PRISMA) guidelines and was registered in the PROSPERO database (CRD420251106228). All European cohort studies comparing the efficacy and adverse events of Axi-cel and Tisa-cel in patients with LBCL were eligible for inclusion. Two authors (Haoxuan Li and Yingchu Liu) independently searched MEDLINE, PubMed, Google Scholar, Scopus, the Cochrane Library, and ClinicalTrials.gov using the following keywords: “Axicabtagene Ciloleucel”, “Tisagenlecleucel”, “Large B-Cell Lymphoma”, “Europe”, and “Comparative study”, combined with the Boolean operators AND/OR. The search was limited to publications from January 2020 to February 2026. In addition to direct database searches, reference lists of eligible articles were manually screened and further analyzed for additional qualifying studies.

### Inclusion and exclusion criteria

Eligibility criteria were developed using the PICOS framework (Population, Intervention, Comparator, Outcomes, Study design). Population (P): European patients with large B-cell lymphoma. Intervention (I): Axi-cel. Comparator (C): Tisa-cel. Outcomes (O): Efficacy and survival endpoints included 3-month OR, 3-month CR, 12-month PFS, and 12-month OS; toxicity endpoints included 12-month NRM, all-grade CRS, grade ≥3 CRS, all-grade ICANS, grade ≥3 ICANS, all-grade neutropenia, grade ≥3 neutropenia, all-grade thrombocytopenia, grade ≥3 thrombocytopenia, all-grade anemia, grade ≥3 anemia, tocilizumab use, and ICU support. Study design (S): non-randomized cohort studies reporting at least three efficacy or toxicity outcomes. Exclusion criteria included: non-English publications, conference abstracts or letters, studies reporting outcomes from CAR-T therapy combined with other treatments, studies in which patients received other CAR-T products (*e.g.*, Lisocabtagene Maraleucel, Brexucabtagene Autoleucel), non-European case-control studies, and animal studies.

### Study selection and data collection

Two authors (Haoxuan Li and Yingchu Liu) independently screened records according to the inclusion and exclusion criteria. Titles and abstracts were first reviewed to identify potentially eligible studies, followed by full-text assessment of each qualifying article. Any discrepancies were resolved through discussion, with each disputed article evaluated systematically against the eligibility criteria. When necessary, a third reviewer (Xinyang Huang) was consulted, or a team meeting was convened to reach consensus.

### Data extraction and management

Data were extracted from eligible studies and entered into a standardized Excel spreadsheet. Key extracted variables included first author, publication year, study design, country, patient demographic characteristics, commonly used hematological baseline parameters, efficacy and survival outcomes, and toxicity outcomes.

### Statistical analysis

Statistical analyses were performed using Review Manager V5.3.1 and R V4.2.2. Results are reported with 95% confidence intervals (CIs). Odds ratios (ORs) were used for dichotomous variables, and weighted mean differences (WMDs) for continuous variables. For studies lacking mean (M) and standard deviation (SD) data, we converted the reported values to mean and SD using the method described by Luo et al. Dichotomous variables were analyzed using the Mantel-Haenszel method, and continuous variables using the inverse-variance method. Given the limited number of included studies and the potential for substantial between-study heterogeneity, all analyses were conducted using a random-effects model. Heterogeneity was assessed using the *I*^2^ statistic, with values of 0%–30% indicating low heterogeneity, 30%–60% moderate, 60%–90% substantial, and 90%–100% considerable. To assess the potential confounding effect of baseline age differences on efficacy and toxicity outcomes, we conducted a random-effects meta-regression using the mean age of each study as the covariate. The model used the log OR of Axi-cel *versus* Tisa-cel for each outcome as the dependent variable, estimated by restricted maximum likelihood (REML), and weighted by the inverse of the standard error (SE). Statistical significance was set at *P* < 0.05. Statistical significance was defined as *P* < 0.05 throughout. Study quality was assessed using the ROBINS-I tool, as all included studies were observational cohort studies. Sensitivity analyses were performed for outcomes with significant heterogeneity to examine the robustness of our conclusions. Given that fewer than 10 studies were ultimately included, statistical power was insufficient to conduct formal publication bias analyses.

## Results

### Baseline characteristics

**Figure 1:**
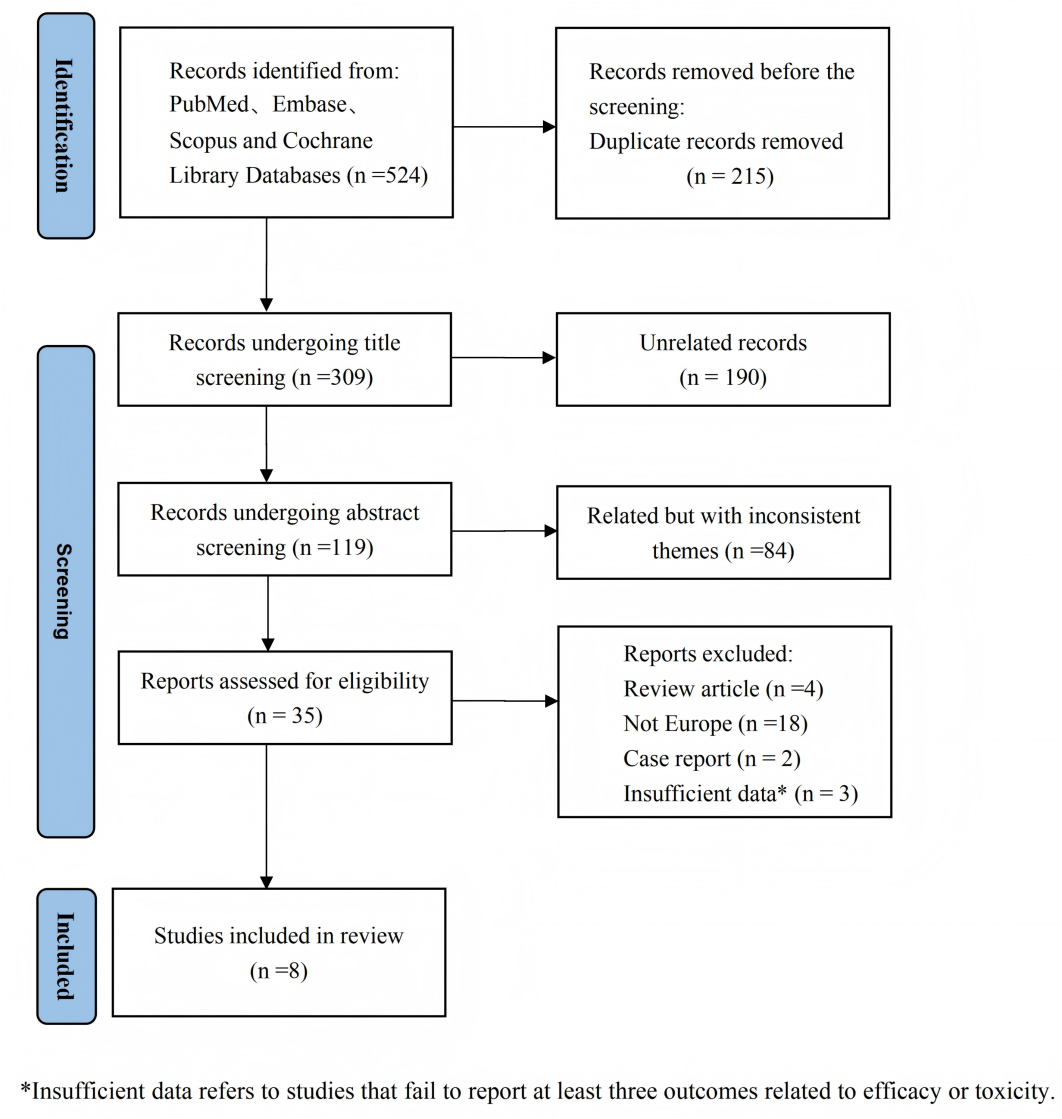
The PRISMA Flowchart.

Based on our search strategy and eligibility criteria, eight studies were identified and included in the meta-analysis (Bachy et al., 2022; Bethge et al., 2022; Brijs et al., 2024; Camacho-Arteaga et al., 2025; Kuhnl et al., 2022; Kwon et al., 2023; Sesques et al., 2020; Stella et al., 2024). Table 1 summarizes the key baseline characteristics of these studies. The studies were published between 2020 and 2025 and covered six European countries: Spain, France, Germany, Belgium, the United Kingdom, and Italy. A total of 2,178 patients were analyzed, of whom 1,179 received Axi-cel and 999 received Tisa-cel. Figure 1 presents the study selection process based on the PRISMA flow diagram. Table 2 presents comparative data on patient demographic and hematological baseline characteristics across the included studies. Our analysis showed that, with the exception of age (*P* < 0.0001), no statistically significant differences were observed between the Axi-cel and Tisa-cel groups for the following baseline variables: sex (*P* = 0.50), prior lines of therapy (*P* = 0.37), prior SCT (*P* = 0.72), Ann Arbor stage I–II (*P* = 0.19), bulky disease (*P* = 0.16), IPI score 0–2 (*P* = 0.54), ECOG performance status 0–1 (*P* = 0.42), LDH >normal (*P* = 0.75), and bridging therapy (*P* = 0.39), indicating good baseline comparability between groups. Regarding age, meta-regression was performed to quantify the impact of between-group age differences on efficacy and toxicity outcomes. The results showed that, with the exception of all-grade thrombocytopenia (*β* = 0.44, *P* < 0.001), age had no significant confounding effect on any other outcome (*P* > 0.05). This suggests that the main conclusions of this study are largely unaffected by baseline age differences and are robust, while the pooled estimate for all-grade thrombocytopenia should be interpreted with caution.

**Table 1:** Baseline characteristics.

Reference	Camacho-Arteaga et al. (2025)		Bachy et al. (2022)		Bethge et al. (2022)		Brijs et al. (2024)		Kuhnl et al. (2022)		Kwon et al. (2023)		Sesques et al. (2020)		Stella et al. (2024)	
Country	Spain		France		Germany		Belgium		UK		Spain		France		Italy	
Study design	Prospective cohort		Retrospective cohort		Retrospective cohort		Retrospective cohort		Prospective cohort		Retrospective cohort		Retrospective cohort		Prospective cohort	
Center type	Multicenter		Multicenter		Multicenter		Single-center		Multicenter		Multicenter		Single-center		Multicenter	
Treatment	Axi-cel	Tisa-cel	Axi-cel	Tisa-cel	Axi-cel	Tisa-cel	Axi-cel	Tisa-cel	Axi-cel	Tisa-cel	Axi-cel	Tisa-cel	Axi-cel	Tisa-cel	Axi-cel	Tisa-cel
Patients	117	55	209	209	173	183	43	36	224	76	152	155	28	33	233	252
Males	68 (58.1%)	33 (60.0%)	121 (57.9%)	126 (60.3%)	120 (69.4%)	116 (63.4%)	NA	NA	143 (63.8%)	42 (55.3%)	89 (58.6%)	97 (62.6%)	16 (57.1%)	24 (72.7%)	154 (66.1%)	153 (60.7%)
Age (years)	56.70 ± 13.50	62.30 ± 13.60	61.15 ± 10.73	63.08 ± 11.09	59.34 ± 11.72	60.26 ± 11.83	NA	NA	56.42 ± 10.82	62.16 ± 9.79	58.57 ± 9.46	60.96 ± 10.00	56.65 ± 11.18	59.64 ± 11.30	56.70 ± 12.10	58.70 ± 10.70
Prior lines of therapy	2.35 ± 0.75	2.65 ± 0.76	2.20 ± 1.09	2.27 ± 1.46	NA	NA	NA	NA	NA	NA	2.17 ± 0.76	2.21 ± 0.94	NA	NA	2.50 ± 0.85	2.44 ± 0.78
Prior SCT	42 (35.9%)	26 (47.3%)	49 (23.4%)	46 (22.0%)	57 (32.9%)	64 (35.0%)	NA	NA	41 (18.3%)	9 (11.8%)	45 (29.6%)	43 (27.7%)	8 (28.6%)	10 (30.3%)	61 (26.2%)	73 (29.0%)
Ann Arbor stage I–II	NA	NA	44 (21.1%)	38 (18.2%)	NA	NA	NA	NA	49 (21.9%)	15 (19.7%)	35 (23.0%)	38 (24.5%)	8 (28.6%)	7 (21.2%)	70 (30.0%)	62 (24.6%)
Bulky disease (longest diameter > 7.5 cm)	NA	NA	39 (18.7%)	45 (21.5%)	NA	NA	NA	NA	71 (31.7%)	10 (13.2%)	40 (26.3%)	35 (22.6%)	NA	NA	88 (37.8%)	77 (30.6%)
IPI score 0–2	NA	NA	88 (42.1%)	72 (34.4%)	81 (46.8%)	104 (56.8%)	NA	NA	114 (50.9%)	35 (46.1%)	73 (48.0%)	70 (45.2%)	NA	NA	132 (56.7%)	133 (52.8%)
ECOG performance status 0–1	NA	NA	178 (85.2%)	173 (82.8%)	146 (84.4%)	154 (84.2%)	NA	NA	202 (90.2%)	69 (90.8%)	144 (94.7%)	144 (92.9%)	21 (75.0%)	22(66.7%)	NA	NA
LDH > Normal	NA	NA	115 (55.0%)	117 (56.0%)	112 (64.7%)	101 (55.2%)	NA	NA	133 (59.4%)	49 (64.5%)	84 (55.3%)	101 (65.2%)	22 (78.6%)	26 (78.8%)	NA	NA

**Notes.**

Data representation of continuous variables mean ± SD SD standard deviation Data representation of discrete variables n (%) SCT stem cell transplantation IPI international prognostic index LDH lactate dehydrogenase NA not available

**Table 2:** The demographics of the studies.

Variable	Number of studies with available data	WMD/OR	95% CI	*P*-value
Male (n)	7	1.07	(0.88, 1.29)	0.50
Age (years)	7	−2.75	(−4.05, −1.45)	<0.0001
Prior lines of therapy (n)	4	−0.07	(−0.21,0.08)	0.37
Prior SCT (n)	7	0.96	(0.79, 1.18)	0.72
Ann Arbor stage I–II (n)	5	1.18	(0.92, 1.50)	0.19
Bulky disease (n)	4	1.36	(0.88, 2.11)	0.16
IPI score 0–2 (n)	5	1.08	(0.84, 1.39)	0.54
ECOG performance status 0–1 (n)	5	1.14	(0.83, 1.54)	0.42
LDH > Normal (n)	5	0.95	(0.70, 1.29)	0.75
Bridging therapy(n)	6	0.86	(0.62, 1.21)	0.39

**Notes.**

WMD weighted mean difference OR odds ratio CI confidence interval SCT stem cell transplantation IPI international prognostic index LDH lactate dehydrogenase

### Quality assessment

**Figure 2:**
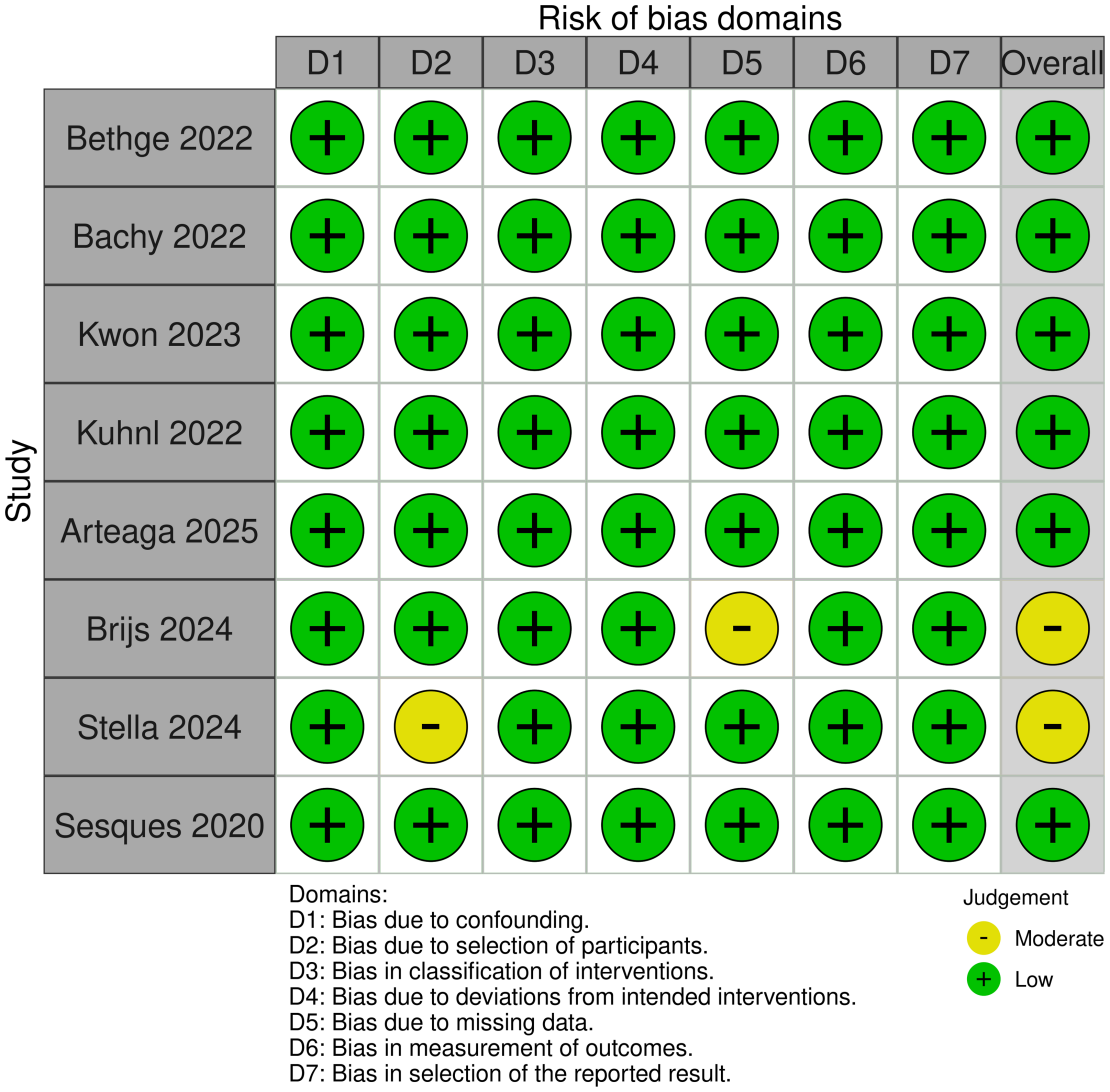
ROBINS-I tool: quality evaluation chart ROBINS-I.

Quality assessment of the included cohort studies was performed using the ROBINS-I tool. The studies by Brijs et al. (2024) and Stella et al. (2024) were rated as having moderate risk of bias, while the remaining studies were rated as low risk (detailed results are shown in Fig. 2). Although these two studies carry a moderate risk of bias, they reported our efficacy and toxicity outcomes of interest in a thorough and comprehensive manner (Table 1), and had relatively large sample sizes, which justified their inclusion. The results related to these studies will be interpreted with caution, with particular attention to their performance in sensitivity analyses and their influence on the overall robustness of our conclusions.

### Efficacy

**Figure 3:**
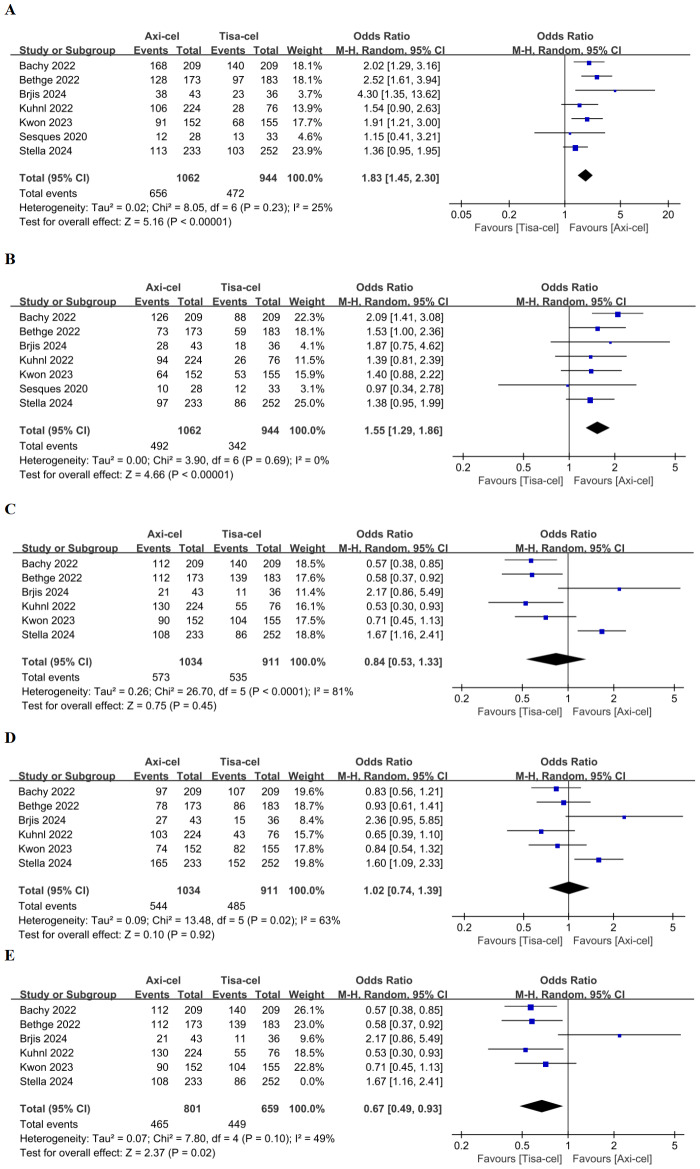
Efficacy related indicators. (A) Forest Plots for 3-month OR; (B) 3-month CR; (C) 12-month PFS with all literature; (D) 12-month OS; (E) 12-month PFS without Stella2024.

Table 3 summarizes the efficacy outcomes of the included studies. Pooled analysis across seven studies showed that, compared with Tisa-cel, Axi-cel was associated with significantly higher 3-month OR rates (OR 1.83, 95% CI [1.45–2.30], *P* < 0.00001) (Fig. 3A) and 3-month CR rates (OR 1.55, 95% CI [1.29–1.86], *P* < 0.00001) (Fig. 3B). However, no significant differences were observed between the two CAR-T products in 12-month PFS (OR 0.84, 95% CI [0.53–1.33], *P* = 0.45) (Fig. 3C) or 12-month OS (OR 1.02, 95% CI [0.74–1.39], *P* = 0.92) (Fig. 3D). Further sensitivity analysis revealed that, after excluding the Stella study, the pooled effect estimate for the highly heterogeneous 12-month PFS (*I*^2^ = 81%) became statistically significant (OR 0.67, 95% CI [0.49–0.93], *P* = 0.02) (Fig. 3E; see Sensitivity Analysis section for details).

### Toxicity

**Figure 4:**
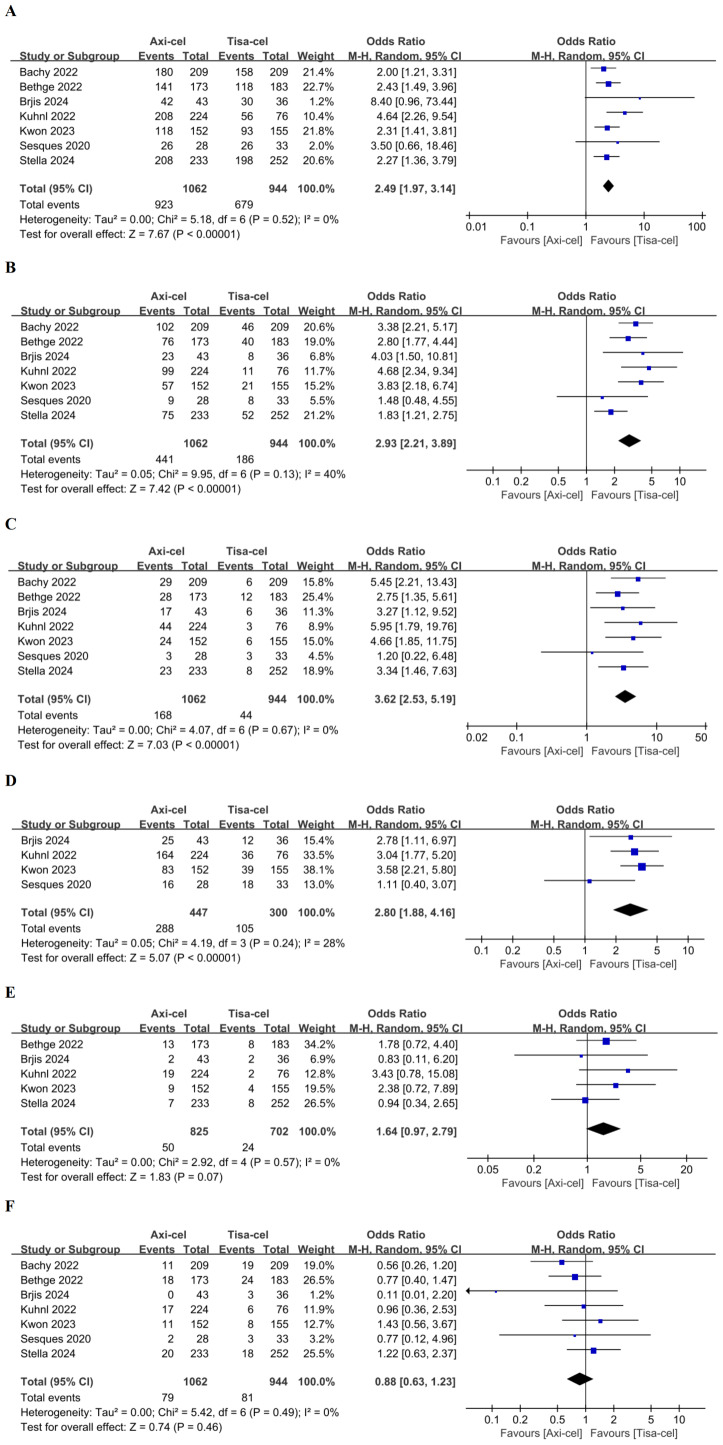
Toxicity outcomes related to cytokine release syndrome, neurotoxicity, and healthcare resource utilization. (A) Forest Plot for all-grade CRS; (B) all grade ICANS; (C) grade ≥ 3 ICANS; (D) Tocilizumab use; (E) 12-month NRM; (F) grade ≥ 3 CRS.

**Figure 5:**
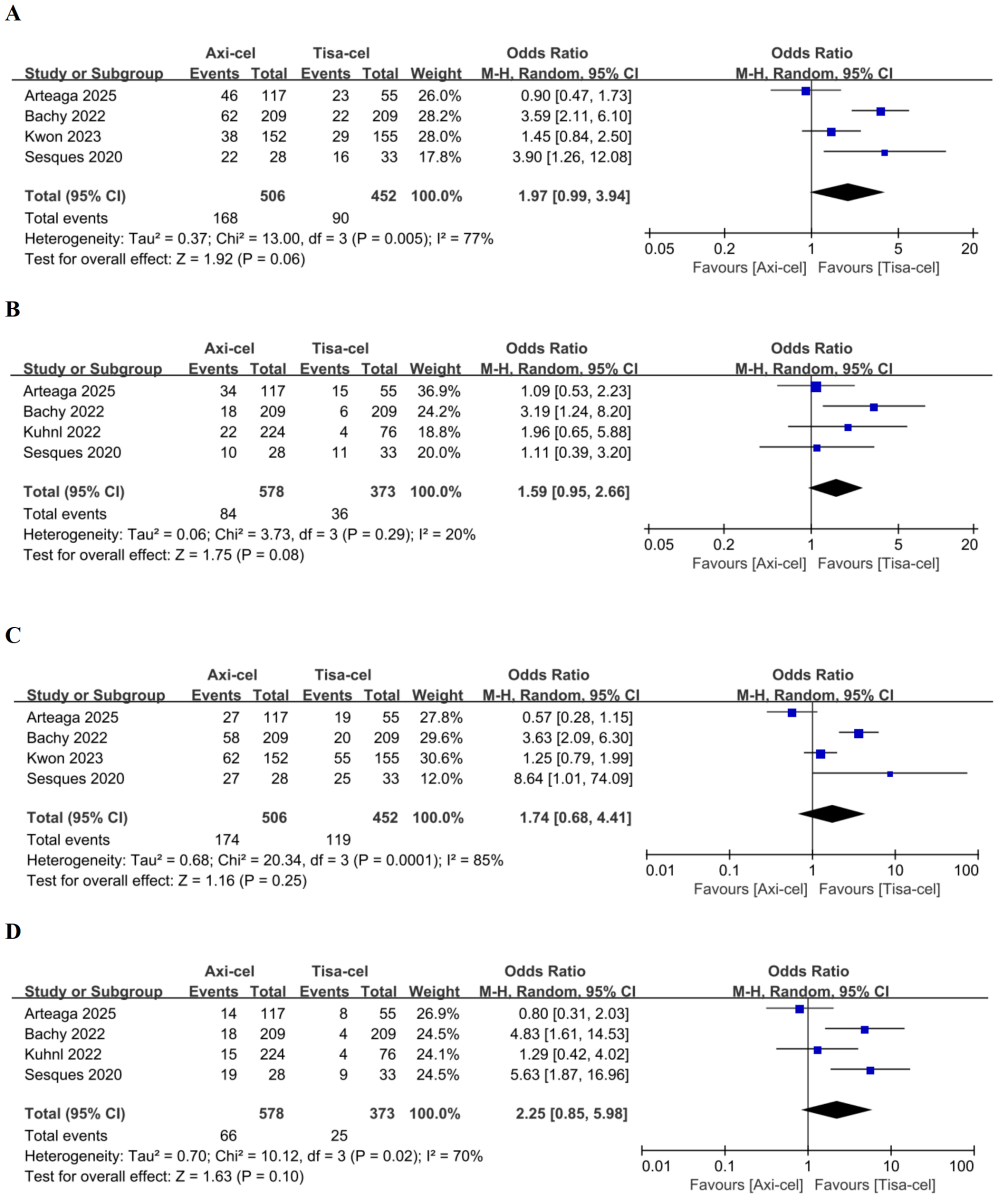
Toxicity outcomes related to neutropenia and thrombocytopenia. (A) All-grade neutropenia; (B) grade ≥ 3 neutropenia; (C) all-grade thrombocytopenia; (D) grade ≥ 3 thrombocytopenia.

**Figure 6:**
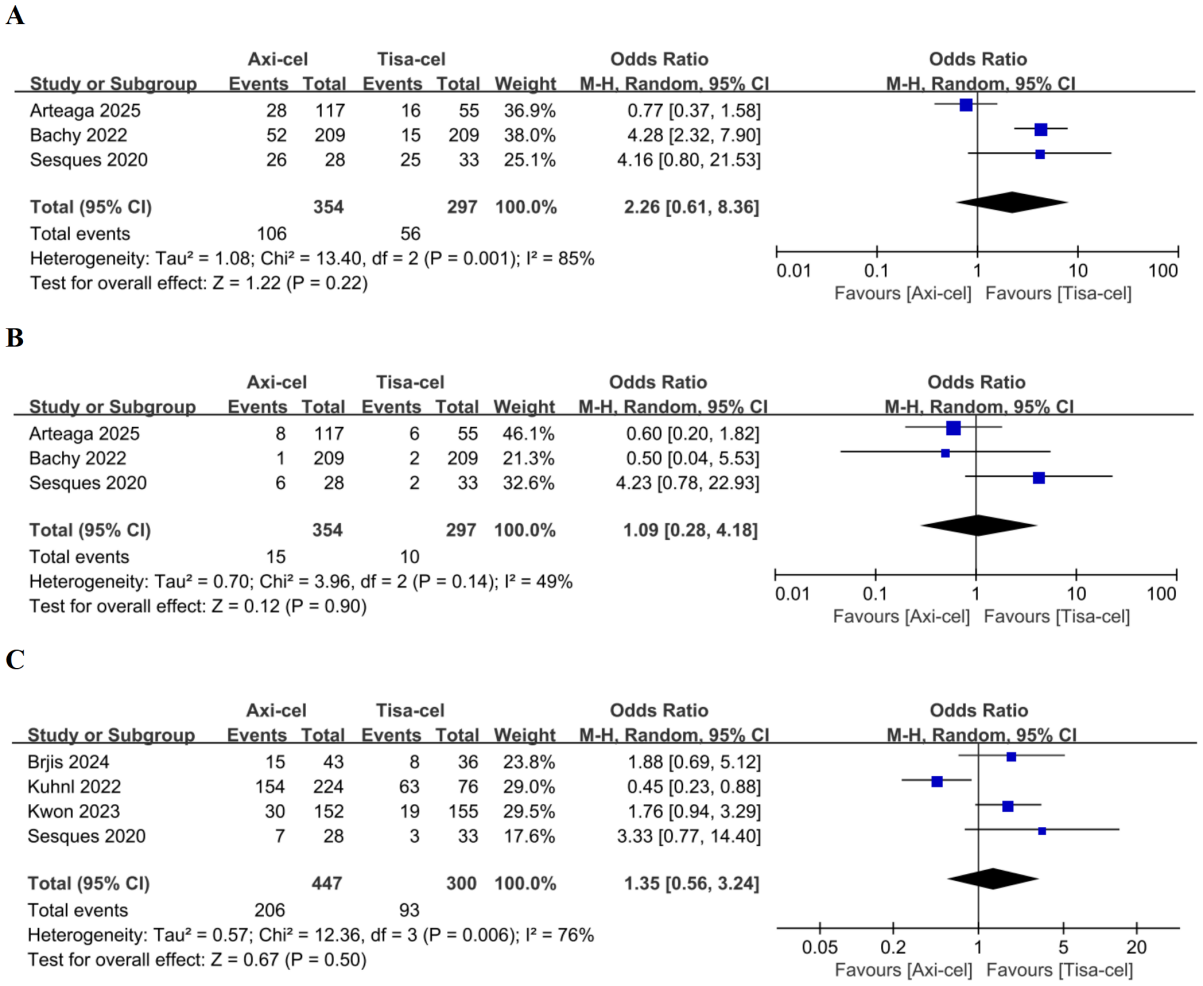
Toxicity outcomes related to anemia and intensive care unit support. (A) All-grade anemia; (B) grade ≥ 3 anemia; (C) ICU support.

Table 4 summarizes the toxicity outcomes across the included studies. Pooled results showed that, compared with Axi-cel, the Tisa-cel group had significantly lower rates of all-grade CRS (OR 2.49, 95% CI [1.97–3.14], *P* < 0.00001) (Fig. 4A), all-grade ICANS (OR 2.93, 95% CI [2.21–3.89], *P* < 0.00001) (Fig. 4B), grade ≥3 ICANS (OR 3.62, 95% CI [2.53–5.19], *P* < 0.00001) (Fig. 4C), and tocilizumab use (OR 2.80, 95% CI [1.88–4.16], *P* < 0.00001) (Fig. 4D). No statistically significant between-group differences were observed for the following toxicity outcomes: NRM at 12 months (OR 1.64, 95% CI [0.97–2.79], *P* = 0.07) (Fig. 4E), grade ≥3 CRS (OR 0.88, 95% CI [0.63–1.23], *P* = 0.46) (Fig. 4F), all-grade neutropenia (OR 1.97, 95% CI [0.99–3.94], *P* = 0.06) (Fig. 5A), grade ≥3 neutropenia (OR 1.59, 95% CI [0.95–2.66], *P* = 0.08) (Fig. 5B), all-grade thrombocytopenia (OR 1.74, 95% CI [0.68–4.41], *P* = 0.25) (Fig. 5C), grade ≥3 thrombocytopenia (OR 2.25, 95% CI [0.85–5.98], *P* = 0.10) (Fig. 5D), all-grade anemia (OR 2.26, 95% CI [0.61–8.36], *P* = 0.22) (Fig. 6A), grade ≥3 anemia (OR 1.09, 95% CI [0.28–4.18], *P* = 0.90) (Fig. 6B), and ICU support (OR 1.35, 95% CI [0.56–3.24], *P* = 0.50) (Fig. 6C).

### Sensitivity analysis

Several outcomes in our meta-analysis exhibited substantial heterogeneity: *I*^2^ = 81% for 12-month PFS, 77% for all-grade neutropenia, 85% for all-grade thrombocytopenia, 85% for all-grade anemia, and 76% for ICU support. To reduce the impact of heterogeneity, we conducted sensitivity analyses using a leave-one-out approach to explore its potential sources. After excluding the Stella study, heterogeneity for 12-month PFS decreased from *I*^2^ = 81% to *I*^2^ = 49%, and the Tisa-cel group showed significantly higher 12-month PFS compared with the Axi-cel group. Further analysis revealed that 75.0% of Tisa-cel-treated patients in the Stella study had Ann Arbor stage III–IV disease, compared with 69.1% in the Axi-cel group, suggesting greater lymph node involvement and more advanced disease in the Tisa-cel group, which may have influenced the 12-month PFS comparison. Sensitivity analyses were also conducted individually for all other efficacy and toxicity outcomes, none of which showed significant changes in heterogeneity, further supporting the robustness of our conclusions for these endpoints.

## Discussion

This is the first systematic review and meta-analysis to compare the efficacy and safety of Axi-cel and Tisa-cel specifically in European patients with LBCL. We found that, within the European context, Axi-cel demonstrated superior short-term treatment efficacy (within three months) compared with Tisa-cel; however, certain intermediate-term efficacy endpoints (at approximately one year) favored Tisa-cel; and the incidences of several adverse events were significantly higher with Axi-cel. For the global population, one meta-analysis and one network meta-analysis have examined the comparative efficacy and safety of Axi-cel and Tisa-cel, with findings regarding short-term efficacy and adverse events consistent with our results. However, these analyses indicated that Axi-cel performed better than Tisa-cel on intermediate-term efficacy outcomes, a discrepancy that may largely reflect differences in patient population characteristics and study design (Bethge et al., 2022; Oluwole et al., 2024).

**Table 3:** Efficacy related indicators.

Reference	Camacho-Arteaga et al. (2025)		Bachy et al. (2022) ^*^		Bethge et al. (2022)		Brijs et al. (2024)		Kuhnl et al. (2022)		Kwon et al. (2023)		Sesques et al. (2020)		Stella et al. (2024)	
Treatment	Axi-cel	Tisa-cel	Axi-cel	Tisa-cel	Axi-cel	Tisa-cel	Axi-cel	Tisa-cel	Axi-cel	Tisa-cel	Axi-cel	Tisa-cel	Axi-cel	Tisa-cel	Axi-cel	Tisa-cel
Patients	117	55	209	209	173	183	43	36	224	76	152	155	28	33	233	252
3-month OR	NA	NA	168 (80.4%)	140 (67.0%)	128 (74.0%)	97 (53.0%)	38 (88.4%)	23 (63.9%)	106 (47.3%)	28 (36.8%)	91 (59.9%)	68 (43.9%)	12 (42.9%)	13 (39.4%)	113 (48.5%)	103 (40.9%)
3-month CR	NA	NA	126 (60.3%)	88 (42.1%)	73 (42.2%)	59 (32.2%)	28 (65.1%)	18 (50.0%)	94 (42.0%)	26 (34.2%)	64 (42.1%)	53 (34.2%)	10 (35.7%)	12 (36.4%)	97 (41.6%)	86 (34.1%)
12-month PFS	NA	NA	112 (53.6%)	140 (67.0%)	112 (64.7%)	139 (76.0%)	21 (48.8%)	11 (30.6%)	130 (58.0%)	55 (72.4%)	90 (59.2%)	104 (67.1%)	NA	NA	108 (46.4%)	86 (34.1%)
12-month OS	NA	NA	97 (46.4%)	107 (51.2%)	78 (45.1%)	86 (47.0%)	27 (62.8%)	15 (41.7%)	103 (46.0%)	43 (56.6%)	74 (48.7%)	82 (52.9%)	NA	NA	165 (70.8%)	152 (60.3%)

**Notes.**

Data representation of discrete variables n (%) OR overall response CR complete remission PFS progression-free survival OS overall survival NA not available

* This study did not specify the response assessment criteria; all other studies used the 2014 Lugano criteria.

Compared with other regions of the world, European populations have distinct characteristics in terms of genetic background, dietary habits, and age structure—factors that are closely linked to LBCL risk and may continuously influence treatment response to CAR-T therapy in this population (Ardisson Korat et al., 2022). Genome-wide association (GWA) analyses have identified the greatest number of genetic susceptibility loci associated with DLBCL risk in individuals of European ancestry (Wang, 2023). Furthermore, the Western European diet, rich in refined carbohydrates and processed and red meat, can promote low-grade systemic chronic inflammation (Juber et al., 2025). Sustained activation of chronic inflammatory signals—including nuclear factor-*κ*B (NF-*κ*B) and signal transducer and activator of transcription 3 (STAT3)—has been shown to be a key driver of the intense proliferation and malignant transformation of B cells in germinal centers (El-Daly et al., 2020; Weniger, Seifert & Küppers, 2025). Additionally, processed meats generate nitrites during curing and preservation, while red meat cooked at high temperatures (*e.g.*, grilling, frying) produces polycyclic aromatic hydrocarbons and heterocyclic amines. These carcinogens, together with the heme iron abundant in red meat, exert direct genotoxic and pro-oxidant effects, potentially promoting malignant B-cell transformation through DNA damage and chronic inflammation (Lee et al., 2024). A large prospective cohort study demonstrated a significant association between refined carbohydrate consumption and increased FL risk (*β* = 0.002, *P* = 0.0002) (Saberi Hosnijeh et al., 2021). On the other hand, owing to relatively well-developed healthcare systems and social welfare programs, European populations have longer average life expectancies and a higher mean age at LBCL diagnosis (Bocean & Vărzaru, 2024; Wéber et al., 2023), and advanced age is an important adverse factor affecting both the efficacy and safety of CAR-T therapy for LBCL (Maiolo et al., 2025; Wudhikarn et al., 2025). In summary, the development of LBCL and the response to CAR-T therapy cannot be generalized from a global perspective when considering the European population. The present study, which focuses specifically on European patients to systematically compare the clinical performance of Axi-cel and Tisa-cel in LBCL, therefore carries important clinical relevance.

Our findings show that Axi-cel was associated with significantly higher 3-month OR and 3-month CR rates compared with Tisa-cel, indicating a short-term efficacy advantage. For OR, a U.S. retrospective cohort study and a global meta-analysis support our conclusion (Gagelmann et al., 2024; Riedell et al., 2022), and a global meta-analysis by Jacobson et al. also supports a higher CR rate with Axi-cel (Jacobson et al., 2024). Weinkove et al. and Zhou et al. demonstrated that, compared with the 4-1BB costimulatory domain used in Tisa-cel, the CD28 costimulatory domain in Axi-cel drives more rapid and potent early T-cell activation and expansion, which underlies its short-term efficacy advantage against LBCL (Weinkove et al., 2019; Zhou et al., 2022). At the molecular level, CD28 signaling rapidly activates the AKT-mTORC1 and ERK1/2 pathways, enhances phosphorylation of key proteins, and efficiently induces expression of factors such as NFAT1, c-Fos, and IL-2, thereby accelerating T-cell proliferation and antitumor immune function (Liang et al., 2024). Mass spectrometry and phosphoproteomics studies have confirmed that T-cell activation induced by CD28/CD3*ζ* signaling is significantly greater in both kinetics and magnitude than that induced by 4-1BB/CD3*ζ* signaling (Philipson et al., 2020). Furthermore, the time from leukapheresis to infusion is generally longer for Tisa-cel than for Axi-cel: one study reported median times of 48.4 days and 30.6 days, respectively (Locke et al., 2025), while a U.S. multicenter study reported median times of 43 days and 35 days, respectively (Deschênes-Simard et al., 2025). Rallon et al., (2026) noted that processing delays may impair the viability and function of *ex vivo* leukocytes, thereby reducing the antihematological efficacy of CAR-T therapy. It is also noteworthy that Axi-cel is typically transduced using a retroviral vector, whereas Tisa-cel uses a lentiviral vector (Locke et al., 2025) and Axi-cel is generally manufactured from fresh leukapheresis products, whereas Tisa-cel tends to use cryopreserved material (Liao et al., 2024). These manufacturing differences may also represent contributing factors to the observed short-term efficacy differences between Axi-cel and Tisa-cel.

**Table 4:** Toxicity related indicators.

Reference	Camacho-Arteaga et al. (2025) ^*^		Bachy et al. (2022)		Bethge et al. (2022)		Brijs et al. (2024)		Kuhnl et al. (2022)		Kwon et al. (2023)		Sesques et al. (2020)		Stella et al. (2024)	
Treatment	Axi-cel	Tisa-cel	Axi-cel	Tisa-cel	Axi-cel	Tisa-cel	Axi-cel	Tisa-cel	Axi-cel	Tisa-cel	Axi-cel	Tisa-cel	Axi-cel	Tisa-cel	Axi-cel	Tisa-cel
Patients	117	55	209	209	173	183	43	36	224	76	152	155	28	33	233	252
12-month NRM	NA	NA	NA	NA	13 (7.5%)	8 (4.4%)	2 (4.7%)	2 (5.6%)	19 (8.5%)	2 (2.6%)	9 (5.9%)	4 (2.6%)	NA	NA	7 (3.0%)	8 (3.2%)
All-grade CRS	NA	NA	180 (86.1%)	158 (75.6%)	141 (81.5%)	118 (64.5%)	42 (97.7%)	30 (83.3%)	208 (92.9%)	56 (73.7%)	118 (77.6%)	93 (60.0%)	26 (92.9%)	26 (78.8%)	208 (89.3%)	198 (78.6%)
Grade ≥ 3 CRS	NA	NA	11 (5.3%)	19 (9.1%)	18 (10.4%)	24 (13.1%)	0 (0.0%)	3 (8.3%)	17 (7.6%)	6 (7.9%)	11 (7.2%)	8 (5.2%)	2 (7.1%)	3 (9.1%)	20 (8.6%)	18 (7.1%)
All-grade ICANS	NA	NA	102 (48.8%)	46 (22.0%)	76 (43.9%)	40 (21.9%)	23 (53.5%)	8 (22.2%)	99 (44.2%)	11 (14.5%)	57 (37.5%)	21 (13.5%)	9 (32.1%)	8 (24.2%)	75 (32.2%)	52 (20.6%)
Grade ≥ 3 ICANS	NA	NA	29 (13.9%)	6 (2.9%)	28 (16.2%)	12 (6.6%)	17 (39.5%)	6 (16.7%)	44 (19.6%)	3 (3.9%)	24 (15.8%)	6 (3.9%)	3 (10.7%)	3 (9.1%)	23 (9.9%)	8 (3.2%)
All-grade neutropenia	46 (39.3%)	23 (41.8%)	62 (29.7%)	22 (10.5%)	NA	NA	NA	NA	NA	NA	38 (25.0%)	29 (18.7%)	22 (78.6%)	16 (48.5%)	NA	NA
Grade ≥ 3 neutropenia	34 (29.1%)	15 (27.3%)	18 (8.6%)	6 (2.9%)	NA	NA	NA	NA	22 (9.8%)	4 (5.3%)	NA	NA	10 (35.7%)	11 (33.3%)	NA	NA
All-grade thrombocytopenia	27 (23.1%)	19 (34.5%)	58 (27.8%)	20 (9.6%)	NA	NA	NA	NA	NA	NA	62 (40.8%)	55 (35.5%)	27 (96.4%)	25 (75.8%)	NA	NA
Grade ≥ 3 thrombocytopenia	14 (12.0%)	8 (14.5%)	18 (8.6%)	4 (1.9%)	NA	NA	NA	NA	15 (6.7%)	4 (5.3%)	NA	NA	19 (67.9%)	9 (27.3%)	NA	NA
All-grade anemia	28 (23.9%)	16 (29.1%)	52 (24.9%)	15 (7.2%)	NA	NA	NA	NA	NA	NA	NA	NA	26 (92.9%)	25 (75.8%)	NA	NA
Grade ≥ 3 anemia	8 (6.8%)	6 (10.9%)	1 (0.5%)	2 (1.0%)	NA	NA	NA	NA	NA	NA	NA	NA	6 (21.4%)	2 (6.1%)	NA	NA
Tocilizumab use	NA	NA	NA	NA	NA	NA	25 (58.1%)	12 (33.3%)	164 (73.2%)	36 (47.4%)	83 (54.6%)	39 (25.2%)	16 (57.1%)	18 (54.5%)	NA	NA
ICU support	NA	NA	NA	NA	NA	NA	15 (34.9%)	8 (22.2%)	154 (68.8%)	63 (82.9%)	30 (19.7%)	19 (12.3%)	7 (25.0%)	3 (9.1%)	NA	NA

**Notes.**

Data representation of discrete variables n (%) NRM non-relapse mortality CRS cytokine release syndrome ICANS immune effector cell-associated neurotoxicity syndrome ICU intensive care unit NA not available

* This study did not specify the CRS/ICANS grading system; all other studies used the ASTCT consensus grading criteria.

However, we found that the 12-month PFS rate was significantly higher in the Tisa-cel group than in the Axi-cel group, with no statistically significant difference in OS between the two groups, suggesting a modest intermediate-term efficacy advantage for Tisa-cel in the treatment of LBCL. Regarding PFS, a global meta-analysis by Gagelmann et al. (2024) and a U.S. single-center retrospective cohort study by Ghanem (2023) are consistent with our conclusion regarding OS, a U.S. eight-center collaborative retrospective cohort study showed similar results (Riedell et al., 2022). Sterner & Sterner (2022) noted that CD28 costimulatory signaling in Axi-cel tends to promote CAR-T cell differentiation toward a central memory phenotype dependent on aerobic glycolysis, which may facilitate its rapid onset of action but also leads to earlier T-cell exhaustion. This may help explain the inferior intermediate-term efficacy of Axi-cel compared with Tisa-cel. However, some studies have reported no difference between the two products in PFS or OS, and further high-quality research is needed (Kwon et al., 2023; Riedell et al., 2022).

Pooled analysis of five studies found no significant difference in 12-month NRM between Axi-cel and Tisa-cel for LBCL treatment, consistent with the cohort study by Kwon et al. (2023) NRM is a composite measure of treatment-related toxicity with a complex composition, and severe infection is one of the most common and prominent drivers of intermediate-term NRM (Bethge et al., 2022). Deschênes-Simard et al. (2025) noted that, despite the more severe CRS/ICANS associated with Axi-cel, the risk of life-threatening severe infections may be comparable between Axi-cel and Tisa-cel, which helps explain the similar 12-month NRM observed with both products. However, some studies have reported significantly higher 12-month NRM with Axi-cel than with Tisa-cel, and further high-quality research is needed to clarify this finding (Gagelmann et al., 2024).

Pooled results from seven studies showed that, compared with Axi-cel, the all-grade CRS rate was significantly lower with Tisa-cel, whereas no significant difference was observed for grade ≥3 CRS. A global meta-analysis by Jacobson et al. (2024) yielded results consistent with ours; by contrast, a global meta-analysis by Meng et al. (2021) reported the opposite finding—that is, a higher CRS rate with Tisa-cel—but these authors acknowledged substantial bias in their use of the Penn grading scale. CRS is a systemic inflammatory response caused by excessive immune activation following immunotherapy or infection (Shah, Soper & Shopland, 2023). The molecular design of Tisa-cel underlies its lower all-grade CRS rate. Compared with the CD28 construct in Axi-cel, the 4-1BB costimulatory domain in Tisa-cel provides a more moderate and sustained T-cell activation, resulting in reduced cytokine release and a lower rate of cytokine storm. For grade ≥3 CRS, however, its occurrence is more heavily influenced by patient-specific factors, clinical management strategies, and dosing approaches rather than inherent differences in CAR-T product design (Banerjee, Fakhri & Shah, 2021; Halford, Anderson & Bennett, 2021; Ortíz-Maldonado et al., 2021). In clinical practice, because grade ≥3 CRS can be life-threatening, its occurrence is often effectively managed by early interventions such as tocilizumab and corticosteroids (Rodrigues Dos Santos, Zanini & Andolfatto, 2024). Through proactive clinical management, the incidence of grade ≥3 CRS can be substantially controlled, which may attenuate or even eliminate differences in grade ≥3 CRS rates between CAR-T products (Topp et al., 2021).

Our meta-analysis showed that the incidences of all-grade ICANS and grade ≥3 ICANS were significantly lower with Tisa-cel than with Axi-cel for LBCL treatment, indicating that Tisa-cel is associated with a lower burden of neurotoxicity (Gagelmann et al., 2024; Han et al., 2024; Shouval et al., 2025; Yamshon et al., 2024). Multiple studies have yielded results consistent with ours, strengthening the reliability of our conclusions. ICANS is a central nervous system inflammatory syndrome triggered by uncontrolled cytokine storm following immunotherapy, characterized by neurological manifestations including aphasia, confusion, memory impairment, and motor deficits; it frequently co-occurs with or follows CRS (Maillie et al., 2025; Nie et al., 2024). The high-level release of pro-inflammatory cytokines such as IL-1 and IL-6 in ICANS damages vascular endothelial cells, disrupts the blood–brain barrier, and directly or indirectly activates endothelial cells, microglia, and other cells in the central nervous system, inducing neuroinflammatory responses and neurotoxic symptoms (Chen et al., 2021; Holtzman et al., 2021). As noted above, the 4-1BB domain in Tisa-cel generally drives a lower overall intensity of CRS induction than Axi-cel. This more moderate and sustained T-cell expansion, along with the relatively lower peak inflammatory burden, is an important factor underlying the lower downstream incidence of ICANS. The reduced ICANS risk associated with Tisa-cel compared with Axi-cel confers multiple benefits: it is associated with lower rates of serious neurological complications such as stroke, intracranial hemorrhage, and cerebral edema; it can alleviate patient anxiety and distress; and it shortens hospital stays and reduces healthcare costs—all of which are advantageous for both individual patients and the broader healthcare system (Lapidus et al., 2022; Sesques et al., 2020).

Regarding commonly assessed complete blood count parameters, our analysis showed no significant difference between Axi-cel and Tisa-cel in the rates of neutropenia, thrombocytopenia, or anemia at either all-grade or grade ≥3 severity. A single-center retrospective study and a meta-analysis have both yielded results consistent with our conclusions, further supporting their reliability (Gagelmann et al., 2024; Wakabayashi et al., 2024). Notably, meta-regression results suggested a trend toward a higher relative risk of all-grade thrombocytopenia with Axi-cel as patient age increased, which may be related to reduced bone marrow reserve and greater susceptibility to CAR-T cell-associated hematological toxicity in older patients (Ullrich et al., 2024). Given the limited number of studies and the high heterogeneity in the meta-analysis for all-grade thrombocytopenia, larger prospective high-quality studies are warranted to clarify the comparative findings.

Pooled results from four studies found that tocilizumab use was significantly lower in the Tisa-cel group compared with Axi-cel, whereas no significant difference was observed between groups in the proportion of patients requiring ICU support. The findings of Gagelmann et al. (2024) and Jain et al. (2022) are consistent with our conclusions, indicating that Axi-cel requires greater healthcare resource utilization in some respects. Tocilizumab, which specifically blocks IL-6 to suppress inflammation (García-Pérez et al., 2025), is particularly suited for the prevention and treatment of CRS and ICANS (Martin et al., 2023). The higher incidence of CRS and ICANS with Axi-cel directly accounts for its more frequent tocilizumab use. Our meta-analysis is currently the only one to compare the proportion of patients requiring ICU support between Axi-cel and Tisa-cel. Similar to the findings for 12-month NRM, the lack of a significant between-group difference in ICU support rates may reflect the effect of rigorous early intervention and clinical management strategies; further high-quality studies are needed to explore this question in depth.

Throughout the course of LBCL treatment, the overall cost of Axi-cel is generally higher than that of Tisa-cel. One cost analysis reported mean per-patient costs of $512,021 for Axi-cel and $450,885 for Tisa-cel (Ghanem, 2023). Hospitalization and neurological events are important contributors to this cost difference. The mean daily hospitalization cost for Axi-cel in LBCL has been reported at $15,214 (Huguet et al., 2021), and Axi-cel infusion typically requires inpatient admission with a significantly longer median length of stay than Tisa-cel (Liao et al., 2024; Riedell et al., 2022) .Moreover, due to the higher incidence of ICANS with Axi-cel, there is a greater need for dedicated nursing care, more intensive neurological monitoring (*e.g.*, continuous EEG), and imaging assessments (*e.g.*, brain MRI) (Maziarz et al., 2022). The mean cost attributable to ICANS events in the Axi-cel group has been reported at $27,223 per patient, significantly higher than the $16,528 per patient reported for Tisa-cel (Badaracco, Gitlin & Keating, 2023). Additionally, approximately 40% of patients treated with Axi-cel have residual neurological deficits requiring long-term rehabilitation support (Bachy et al., 2022), further adding to the financial burden. Therefore, the selection of a CAR-T regimen for LBCL requires individualized weighing of the efficacy-toxicity-cost triad (Liao et al., 2024).

As with other retrospective studies, a limitation of this analysis is its retrospective design. Although overall baseline comparability between the Axi-cel and Tisa-cel groups was good, residual selection bias cannot be excluded. To quantitatively address the important confounding effect of age, we conducted meta-regression, which enhances the credibility of our results. However, because meta-regression is based on study-level rather than individual patient-level data, it cannot fully exclude the possibility of residual confounding. Additionally, two studies rated as moderate risk of bias (Brijs et al., 2024 and Stella et al., 2024) were included; although sensitivity analyses were performed to mitigate this issue, potential unmeasured confounding factors remain a concern. Furthermore, due to the limited number of eligible studies, further geographic stratification within Europe and subgroup analyses by population baseline characteristics were not feasible, which limits exploration of sources of heterogeneity. Finally, evaluation of the efficacy and safety of Axi-cel and Tisa-cel for LBCL should not be confined to these short- and intermediate-term outcomes within one year; longer-term survival and toxicity data are essential, but were not available from the included studies, precluding a comprehensive assessment of long-term differences. In sum, further high-quality research is needed.

## Conclusion

This study systematically analyzed eight independent cohort studies conducted in Europe, encompassing a total of 2,178 patients. These studies offer a broad European perspective, with data from six countries: Spain, France, Germany, Belgium, the United Kingdom, and Italy. Our findings indicate that, for European patients with LBCL receiving CAR-T therapy, Axi-cel achieves superior short-term efficacy (3-month OR and 3-month CR) compared with Tisa-cel, but is inferior in certain intermediate-term efficacy endpoints (12-month PFS), and is associated with higher rates of toxicity and adverse events (all-grade CRS, all-grade ICANS, grade ≥3 ICANS) and greater healthcare resource utilization in some respects (tocilizumab use). To further refine the efficacy and safety conclusions of this study and to better evaluate the long-term outcomes of Axi-cel and Tisa-cel in LBCL, future randomized controlled trials with extended follow-up periods are needed.

## Supplemental Information

10.7717/peerj.21410/supp-1 Supplemental Information 1 PRISMA checklist.

10.7717/peerj.21410/supp-2 Supplemental Information 2 Raw data.

## Additional Information and Declarations

### Data Availability

The following information was supplied regarding data availability: This is a Systematic review/Meta-analysis.
